# Diabetic Foot: Infections and Outcomes in Iranian Admitted Patients

**DOI:** 10.5812/jjm.11680

**Published:** 2014-07-01

**Authors:** Azar Hadadi, Houra Omdeh Ghiasi, Mahboubeh Hajiabdolbaghi, Majid Zandekarimi, Reza Hamidian

**Affiliations:** 1 Department of Internal Medicine, Research Development Center, Sina Hospital, Tehran University of Medical Sciences, Tehran, IR Iran; 2 Sina Hospital, Tehran University of Medical Sciences, Tehran, IR Iran; 3 Department of Infectious Diseases, Iranian Research Center for HIV and AIDS, Emam Khomeini Hospital, Tehran University of Medical Sciences, Tehran, IR Iran; 4 Zabol University of Pharmacology, Zabol, IR Iran; 5 Research Development Center, Sina hospital, Tehran University of Medical Sciences, Tehran, IR Iran

**Keywords:** Diabetic Foot, Infection, Inpatients, Outcome Assessment

## Abstract

**Background:**

Diabetes mellitus (along with its complications) has become a global problem. Diabetic foot infection, among the most common complications, is responsible for 40 to 50% of foot amputations. Antibiotic-resistant microorganisms, however, have compromised empiric therapy in the infected patients.

**Objectives:**

The current study aimed to determine the most common microorganisms involved in diabetic foot infection in order to minimize the failure of antibiotic therapy and the risk of developing complications.

**Patients and Methods:**

All patients with diabetic foot infection admitted to the infectious diseases, surgery and endocrinology wards of two teaching hospitals from 2007 to 2010 (n = 196) were recruited. In this retrospective study, demographic characteristics, type of lesions, history of hospitalization/antibiotic therapy, isolated microorganisms, clinical complications, administered treatment (medical or surgical) and outcome were recorded.

**Results:**

Patients’ mean age was 60.84 (± 10.30) years. Totally, 113 (57.65%) of the patients were male and 83 (42.35%) were female. According to Wagner’s grading, deep ulcers with/without osteomyelitis accounted for the majority of lesions. A single microorganism was isolated (most common: Escherichia coli, Staphylococcus aureus and Klebsiella spp.) from 81 of the patients (80.20%); while for the remaining polymicrobial infection was reported. Isolated pathogens showed no significant correlation with duration of diabetes, type of the lesions (P = 0.13) and history of hospitalization (P = 0.61). The majority of patients (n = 118, 60.20%) were treated surgically; however 11 patients expired due to sepsis. Amputation (most common at toes and below the knee) was performed for 89 patients (45.40%). The response rate to medical treatment was 31.6% for single-pathogen and 10% for polymicrobial infection (with a 30% mortality rate).

**Conclusions:**

Physicians are recommended to take microbiological cultures before starting empirical therapy recommended to cover Gram-negative microorganisms in order to lower the risk of antibiotic resistance.

## 1. Background

As a globally widespread disease with an increasing incidence, diabetes mellitus has afflicted 150,000,000 people across the world according to the World Health Organization (WHO); and this will be doubling by 2025, (1). Not only the diabetes itself, but also its complications impose a heavy health and economic burden on the societies and health systems. Infections and ulcers accompanied by neuropathy and arteriovenous abnormalities in the foot of patients with diabetes, referred as diabetic foot, are among the most common complications, (2, 3). Diabetic foot infection, also considered as the most important cause of hospitalization in patients with diabetes accounted for 20% of inpatient admissions in North America is responsible for a large number of foot amputations worldwide, (3, 4). Recent studies have estimated that more than 1.5 million Iranians have diabetes, and 7.5% of them are diagnosed with diabetes mellitus type 2, (5); thus diabetic foot infection is a common problem in Iran as well.

Different microorganisms are isolated from diabetic foot infections, based on severity and depth of ulcers. For instance, Gram-positive cocci are the most common germs in superficial ulcers, while anaerobic bacteria are mostly found in deeper lesions (4). The presence of different microorganisms along with increasing resistance to antibiotic therapy has compromised the empiric therapy in diabetic foot infection.

## 2. Objectives

The current study aimed to determine the most common microorganisms responsible for diabetic foot infections in order to minimize the failure of antibiotic therapy and the risk of developing complications (including amputation) in a group of Iranians.

## 3. Patients and Methods

This retrospective cross-sectional multicenter study was conducted in two teaching hospitals affiliated to Tehran University of Medical Sciences, Tehran, Iran. All patients with diabetic foot admitted to the infectious diseases, surgery and endocrinology wards of these two hospitals from 2007 to 2010 were recruited. The diagnosis was made based on WHO criteria for diabetes mellitus diagnosis (approved by American Diabetes Association), (5). The Ethics Board Committee of Tehran University of Medical Sciences approved the study, in conformity to the Declaration of Helsinki. The committee also waived the need for “informed consent” because all subjects had declared the permission for using their data on condition of anonymity at admission.

The patients' demographic characteristics, type of diabetic foot lesions, history of hospitalization, history of antibiotherapy, frequency of isolated microorganisms (according to microbiological cultures), clinical complications, administered treatment (medical or surgical), and relevant outcome were extracted from medical records. The surgical procedures mainly adopted in these patients included abscess drainage, debridement and amputation.

The specimens were checked for aerobic and anaerobic microorganisms through streak culturing them on Eosin methylene blue (EMB), Blood Agar (BA) and chocolate Agar (CA) (Asan Pharmaceutical Co. Ltd. South Korea). Plates were incubated overnight at an incubation temperature of 37°C. In case any bacteria grew on the plates, the specimens would be screened by Gram stain for the presence of multiple types of organisms. Then, the antibiogram was conducted on Muller Hinton Agar using the CLSI disk-diffusion method.

### 3.1. Statistical Analysis

The frequency of characteristics was presented as number (percentage), and quantitative results as mean (± standard deviation). Chi-square test was applied to compare qualitative characteristics and Student t-test was applied for quantitative ones; the quantitative characteristics that did not have normal distribution, however, were compared with Mann-Whitney test. Response to treatment was determined by means of logistic regression analysis. All statistical calculations were done with SPSS ver. 17 (IBM, USA). P value < 0.05 was considered significant.

## 4. Results

One hundred and ninety six patients with the mean age of 60.84 (± 10.30) years were recruited. They included 113 male (57.65%) and 83 female (42.34%), and among them, 34 patients (17.34%) had type I and 162 patients (82.65%) lived with type II diabetes. The mean duration of diabetes mellitus and diabetic foot ulcers were 18.11 (± 10.03) years and 48.98 (± 63.60) days respectively. Fifty-six patients (28.57%) had already received antibiotics before admission. Previous history of hospitalization was reported in 47 patients (23.98%). Information about types of lesions according to Wagner’s grading is summarized in Table 1.

**Table 1.** Frequency of Lesions in Diabetic Foot Infection in Accordance with Wagner Grading System

Wagner’s Grade	Signs	No. (%)
**0**	No ulcer in a high risk foot	0 (0.0)
**1**	Superficial ulcer involving the full skin thickness	23 (11.79)
**2**	Deep ulcer penetrating to ligaments/muscle, but no bone involvement or abscess formation	56 (28.71)
**3**	Deep ulcer with cellulitis or abscess formation, often with osteomyelitis	93 (4769)
**4**	Localized gangrene	23 (1179)
**5**	Extensive gangrene involving the whole foot	10 (5.12)

Table 2 outlines the results of 119 microbiological cultures (51.53%). A single microorganism was isolated from 81 patients (80.20%), whereas the other 20 patients (19.8%) were diagnosed with polymicrobial infection. Escherichia coli, *Staphylococcus aureus* and *Klebsiella* spp. formed the most common pathogens found in the cultures. Taking type of lesion into account, *S. aureus*, *Streptococcus* species, and *E. coli* were the most frequent microorganisms in abscess, and *S. aureus*, *Streptococcus* species, and *Klebsiella* spp. were more common in oozing ulcers.

Isolated pathogens showed no significant correlation with duration of diabetes (P = 0.13), duration of foot lesions (P = 0.13), type of lesions (P = 0.20) and history of hospitalization for diabetic food infection (P = 0.61). Similarly, the relationship of single or polymicrobial infection with type of lesions (P = 0.10) and history of hospitalization for diabetic food infection (P = 1.00) was insignificant, on the contrary, the association between isolated pathogen and patients’ outcome at the time of discharge was statistically significant (P < 0.001). The polymicrobial infections led to worse outcome at the time of discharge (P < 0.001).

The majority of patients (n = 118, 60.20%) were treated surgically and only 78 patients (39.80%) benefited from mere antibiotic therapy. Amputation was performed for 89 patients (45.40%). The level of amputation ranged from toes (n = 47) to below the knee (n = 36), distal of metatarsus (n = 9) and above knee (n = 8). In the meantime, 25 patients (12.1%) benefitted from debridement. The response rate to medical treatment was 31.6% for single pathogen infection vs. 10% for polymicrobial infection. A 30% mortality rate was observed in the latter group. Eleven patients also expired due to sepsis. Table 3 outlines the outcome of treatment at the time of hospital discharge. This table is adjusted according to patients’ microbiological cultures; therefore, the numbers in categories are less than the relevant total number of patients. Treatment failure was calculated as a total of lack of response to antibiotherapy and/or surgical intervention plus discharge against medical advice.

**Table 2.** Frequency of Microorganisms Isolated From 119 Microbiological Cultures of Diabetic Foot Infection

Microorganism	No. (%)
***E. coli***	34 (28.57)
***Staphylococcus aureus***	25 (21.00)
***Klebsiella*** ** spp.**	17 (14.28)
***Pseudomonas*** ** spp.**	10 (8.40)
***Acinetobacter*** ** spp.**	5 (4.20)
***Proteus*** ** spp.**	5 (4.20)
***Streptococcus *** **species**	5 (4.20)
***Citrobacter*** ** spp.**	4 (3.36)
***Stenotrophomonasmaltophilia***	4 (3.36)
***Enterococcus *** **spp.**	4 (3.36)
***Edwardisiella*** **spp.**	3 (2.52)
***Providencia*** ** spp.**	3 (2.52)
**Total**	119 (100)

**Table 3.** The Outcomes of Medical/Surgical Treatment of Diabetic Foot Infection According to the Microbiological Cultures at the Time of Hospital Discharge ^a,b^

Culture	Treatment Failure	Death Due to MI	Death Due to Sepsis	Recovery After Surgery	Recovery After Medical Therapy
**Positive (n = 101)**	9 (8.91)	2 (1.98)	7 (69.30)	60 (59.40)	23 (22.77)
**Negative (n = 95)**	20 (21.05)	0 (0.00)	2 (2.10)	34 (35.79)	39 (41.05)
**Total sum (n = 196)**	29 (14.79)	2 (1.02)	9 (4.59)	94 (47.96)	62 (31.63)

^a^ Abbreviation: MI, myocardial infarction.

^b^ Values are shown as No. (%).

## 5. Discussion

Diabetic foot infection spans from local to necrotizing and life-threatening infections between malleoli and toes, (6). It is the second leading cause of lower limb amputation in North America, (4). Although the prevalence of diabetes type II is reported 7% to 8% for major cities of Iran, Southern Iran is reported to have remarkably higher rate (up to 17%). Recent estimates show that diabetic foot infections occur in 3% of the Iranian population, (3), indicating that approximately 225,000 patients with diabetic foot complications impose a heavy burden on the health system in Iran. Published literature within the past decades have always considered *S. aureus* as the pathogen most likely found in diabetic foot lesions, (4, 7-13); while *E. coli* was the most frequent microorganism found in the present study. 

A published report from Malaysia has also accorded with the same results, stating Gram-negative pathogens (including *Proteus* spp., *Pseudomonas aeruginosa*, *Klebsiella* *pneumonia* and *E. coli*) were the most common isolated microorganisms (52%), (14). This comes when according to another study from Iran, *E. coli* stood as the second most common pathogen with minor discrepancy behind *S. aureus* (15). The prevalence of polymicrobial infections in this study was also remarkably higher than that of the current study (51% vs. 19.8%) (15). The majority of the current study patients had received empiric antibiotic therapy before admission; this may explain the differences noted in the isolated microorganism of the present study and the previous ones. On the contrary to current report, Pittet et al. reported that osteomyelitis, deep tissue infections, and gangrene as the most common lesions in patients with diabetic foot infection, (13). Morales Lozano et al. similarly reported osteomyelitis in 79.5% of their patients (16). The lower prevalence of osteomyelitis in the current study could be due to the fact that advanced diagnostic measures were not applied in the study and the diagnosis was made based on clinical findings. Alavi et al., however, reported a single microorganism, mainly * S. aureus,* as the most frequently isolated bacteria from diabetic foot patients (17).

The current study failed to report any relationship between the patient’s outcome and demographic characteristics, history of hospitalization, duration of diabetes mellitus, neutrophil count and the anatomic site of foot lesions. This comes while in another study, the complication of treatment of diabetic foot infection depended on depth of ulcer, presence of ischemia and severity of glycemic control (18). The patients’ age, gender, type and duration of diabetes and the anatomic site of ulcers did not correlate with the outcome (18). Similarly, Yekta et al. reported the association between the patients’ characteristics and his/her outcomes, stating that those with amputation were significantly older, - less educated, had longer duration of diabetes and had a poor glycemic control (3).

The association between BMI and amputation shown in other studies (3, 19, 20), remained unproved in the current work. Correspondingly, while another study in Iran emphasized the correlation between patients’ gender and amputation (21), the current study could not confirm such an association. Such a correlation, however, was not reported by Li et al. in their study on a population with a 21.8% rate of amputation in China (22).

Amputation rate in the current study not only was higher than that of Yekta et al. (3) but also outweighed a decreasing rate of 40% to 12% by Larijani et al. who took a 22-year trend into account (23). As an implicit finding, slight complications were observed when more microbiological cultures were requested by the physician. Therefore, the treating physicians are recommended to send more samples in shorter time intervals to better diagnosis and choice of antibiotic.

### 5.1. Limitations:

Considering the fact that the data were extracted from the patients’ medical records, missing some data was inevitable. It should be stressed that the results of the baseline culture of some patients were not available; this could explain some of the discrepancy noted between current study findings and those of previous researches. Moreover, since anaerobic culture was not accessible in the hospitals, anaerobic pathogens were not studied in diabetic foot lesions. The diagnosis of osteomyelitis in the current study was based on imaging reports rather than bone biopsy, which is a more definite diagnostic method.

The high rate of amputation noted in the current study could be contributed to the fact that the majority of the patients were referred to the centers late. Physicians are recommended to take microbiological cultures before starting empirical antibiotic therapy, which is recommended to cover Gram-negative microorganism, to lower the risk of experiencing antibiotic resistance. Educating the patients and asking them to visit the diabetic foot clinic more frequently could lower the complication rate in these patients.
